# Dynamic acousto-optic control of a strongly coupled photonic molecule

**DOI:** 10.1038/ncomms9540

**Published:** 2015-10-05

**Authors:** Stephan Kapfinger, Thorsten Reichert, Stefan Lichtmannecker, Kai Müller, Jonathan J. Finley, Achim Wixforth, Michael Kaniber, Hubert J. Krenner

**Affiliations:** 1 Lehrstuhl für Experimentalphysik 1 and Augsburg Centre for Innovative Technologies (ACIT), Universität Augsburg, Universitätsstrasse 1, 86159 Augsburg, Germany; 2 Nanosystems Initiative Munich (NIM), Schellingstrasse 4, 80799 München, Germany; 3 Walter Schottky Institut and Physik Department, Technische Universität München, Am Coulombwall 4, 85748 Garching, Germany; 4 E. L. Ginzton Laboratory, Stanford University, Stanford, California 94305, USA; 5 Center for NanoScience (CeNS), Ludwig-Maximilians-Universität München, Schellingstrasse 4, 80799 München, Germany

## Abstract

Strongly confined photonic modes can couple to quantum emitters and mechanical excitations. To harness the full potential in quantum photonic circuits, interactions between different constituents have to be precisely and dynamically controlled. Here, a prototypical coupled element, a photonic molecule defined in a photonic crystal membrane, is controlled by a radio frequency surface acoustic wave. The sound wave is tailored to deliberately switch on and off the bond of the photonic molecule on sub-nanosecond timescales. In time-resolved experiments, the acousto-optically controllable coupling is directly observed as clear anticrossings between the two nanophotonic modes. The coupling strength is determined directly from the experimental data. Both the time dependence of the tuning and the inter-cavity coupling strength are found to be in excellent agreement with numerical calculations. The demonstrated mechanical technique can be directly applied for dynamic quantum gate operations in state-of-the-art-coupled nanophotonic, quantum cavity electrodynamic and optomechanical systems.

## Abstract (web summary)

Dynamic control of components is required for large-scale quantum photonic networks. Here, Kapfinger *et al*. show dynamic control of the interaction between two coupled photonic crystal nanocavities forming a photonic molecule. Tuning is achieved by using an electrically generated radio frequency surface acoustic wave.

## Graphical abstract

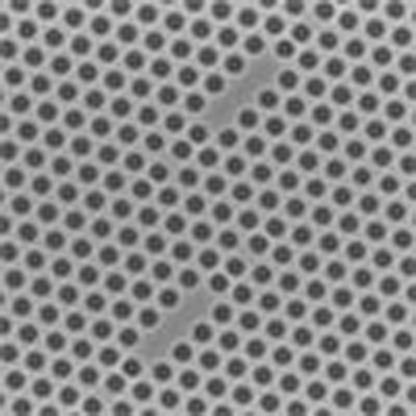

Two-dimensional photonic crystal membranes provide a versatile planar architecture for integrated photonics to control the propagation of light on a chip using high-quality optical cavities, waveguides, beamsplitters or dispersive elements^1^. When combined with highly nonlinear quantum emitters, quantum photonic networks^2^,^3^ operating at the single-photon level^4^ come within reach. Towards large-scale quantum photonic networks^5^,^6^, selective dynamic control of individual components and deterministic interactions between different constituents are of paramount importance^7^. This indeed calls for switching speeds ultimately on the system's native timescales to enable Landau–Zener non-adiabatic control schemes^8^,^9^. For example, manipulation via electric fields or all-optical means have been used for switching in nanophotonic circuits^10^,^11^ and cavity quantum electrodynamics studies^12^,^13^,^14^.

Here, we demonstrate dynamic control of the coherent interaction between two coupled photonic crystal (PhC) nanocavities forming a photonic molecule (PM)^15^,^16^,^17^. By using an electrically generated radio frequency (RF) surface acoustic wave (SAW), we achieve optomechanical tuning^18^. SAWs have been used to dynamically control nanophotonic^18^,^19^,^20^, plasmonic^21^,^22^,^23^, integrated optical devices^24^,^25^ and quantum dot (QD) emitters^26^,^27^,^28^,^29^ at frequencies up to several GHz. The tuning range of our method is sufficiently large to compensate for the inherent fabrication-related cavity mode detuning and the operation speed exceeds that of typical^30^ and downscaled^31^ resonant mechanical approaches. Our findings open a route towards nanomechanically gated protocols^9^, which hitherto have inhibited the realization in all-optical schemes^32^,^33^. Moreover, the mechanical nature of the SAW makes them ideally suited to generate tailored classical phonon fields^34^. The GHz frequencies accessible by SAWs perfectly match that of the mechanical modes in membrane-type PhC photonic^35^ and optomechanical cavities^36^. Thus, our results pave the way towards native mechanical control in these scaled on-chip optomechanical systems.

## Results

### Implementation of acoustically tunable PM

**Figure 1:**
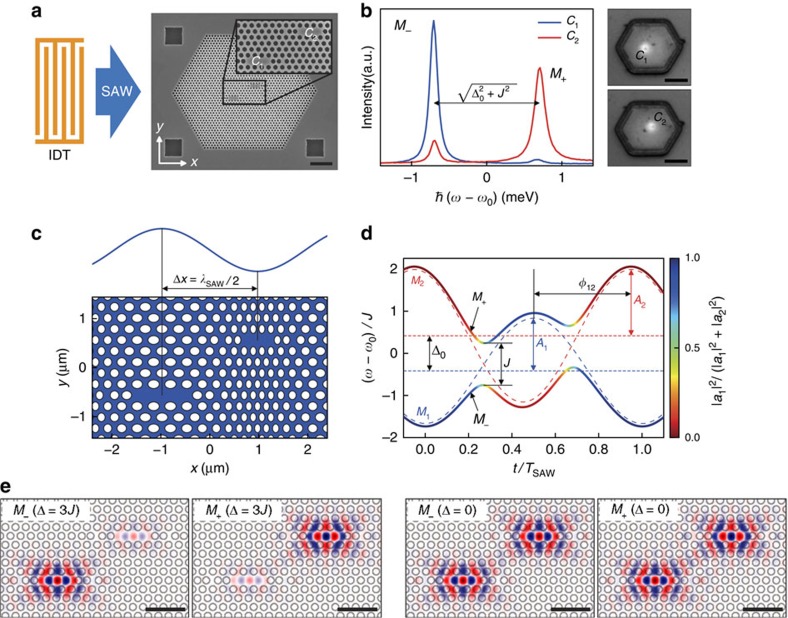
PM with acousto-optic tuning mechanism. (**a**) Scanning electron micrograph of the PM and schematic of the device layout. The IDT launches a SAW that propagates across the PM. Scale bar, 2 μm (IDT not to scale). (**b**) PL spectra showing the two fundamental modes of the PM and optical microscope images of the excitation laser spot. The blue and the red curves were obtained by exciting on cavity *C*_1_ and *C*_2_, respectively. Owing to a finite static detuning Δ_0_>*J*, the modes are confined within their respective cavities. The centre energy is *ω*_0_=1.260 eV. The cavities can be spatially resolved in our optical set-up, allowing selective excitation of the individual cavities. Scale bars, 5 μm. (**c**) Acousto-optic tuning. The resonance frequencies of the cavities are modulated by the SAW-induced strain field (amplitude exaggerated). Setting half the SAW wavelength equal to the cavity separation, the cavities can be tuned relative to each other, giving rise to a time-dependent detuning Δ(*t*)=Δ_0_+Δ_mod_ sin (*ω*_SAW_*t*). (**d**) Evolution of the modes over one SAW period. The dashed lines represent the single-cavity modes *M*_1_ and *M*_2_, and the solid lines represent the normal modes *M*_+_ and *M*_−_. The colour scale corresponds to the decomposition into single-cavity modes, with green denoting the symmetric and antisymmetric superposition on resonance. The static detuning Δ_0_ and the coupling strength *J* are intrinsic parameters of the PM. The tuning amplitudes *A*_1,2_ and the phase shift *φ*_12_ are determined by the amplitude and wavelength of the SAW, respectively. (**e**) FDTD simulations of the mode profiles (*E*_*y*_). For the case of detuned cavities (Δ=3*J*), the modes are localized within the individual cavities. On resonance (Δ=0), one obtains the symmetric (bonding) and the antisymmetric (antibonding) mode. Scale bar, 1 μm.

The on-chip PM studied here consists of two L3-type missing hole cavities^37^, labelled *C*_1_ and *C*_2_, defined in a two-dimensional PhC membrane. Its static coupling strength *J* is known to depend exponentially on the separation between the two cavities^17^. In our sample, the cavities are offset symmetrically by *d*=5 lattice constants (*a*=260 nm) along each primitive direction of the PhC lattice (Supplementary Fig. 1), as can be seen in the scanning electron micrograph in Fig. 1a. The finite coupling strength *J* leads to the formation of two normal modes; a bonding mode *M*_−_ and an antibonding mode *M*_+_. Note that the mode indices in *M*_±_ refer to the respective normal mode frequencies *ω*_±_, and thus are opposite to the spatial symmetry of the mode. In the most general case, the two cavities *C*_1_ and *C*_2_ exhibit resonance frequencies *ω*_1_ and *ω*_2_, split by a finite detuning Δ=*ω*_2_−*ω*_1_. Thus, the normal mode frequencies can be expressed by:


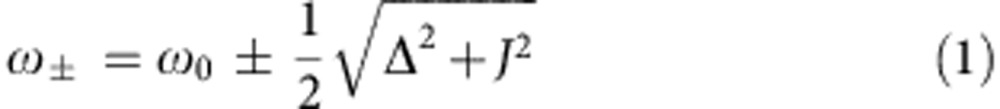


with *ω*_0_=(*ω*_1_+*ω*_2_)/2 being the centre frequency. In Fig. 1b, we present two emission spectra (see Methods section) recorded for spatially exciting either *C*_1_ (blue) or *C*_2_ (red) of a typical PM, denoted as PM1. For PM1, each cavity exhibits a distinct single mode, split by 
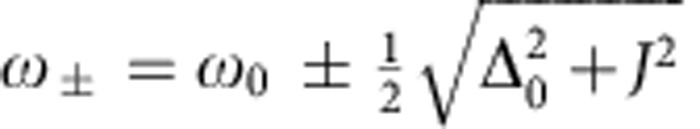
, with Δ_0_ being the static detuning. Such distinct and localized mode patterns are found for the majority of the PMs. This suggests that Δ_0_>*J*, and that the inter-cavity coupling is strongly suppressed. Therefore, as-fabricated and nominally identical cavities are only weakly interacting due to Δ_0_≠0, which arises from inevitable imperfections during nanofabrication. To overcome this fundamental limitation and achieve dynamic control of the individual cavity resonances, we use an optomechanical approach using RF SAWs^18^,^19^. As indicated in the schematic illustration of Fig. 1a, these quasi-monochromatic acoustic phonons are generated by an interdigital transducer (IDT; see Methods section), propagate along the *x* axis of the PM and dynamically deform the individual cavities. The amplitude of this mechanism, ℏ*A*>1 meV, exceeds both the static detuning, Δ_0_, and the coupling strength, *J*, which enables us to dynamically tune the PM completely in and out of resonance. For our chosen geometric arrangement, the cavities are separated by a distance Δ*x* along the SAW propagation direction, the modulations of *ω*_1_ and *ω*_2_ are phase shifted (in unit radians) by *φ*_12_=Δ*x* × *ω*_SAW_/*c*_SAW_, with *ω*_SAW_ and *c*_SAW_ being the SAW angular frequency and speed of sound, respectively. We deliberately set the acoustic wavelength, *λ*_SAW_, such that it is commensurate with the spatial separation of the two cavity centres 2Δ*x*=*λ*_SAW_. As illustrated in the schematic in Fig. 1c, for the selected phase, the maximum (minimum) of the SAW expands *C*_1_ (compresses *C*_2_), which in turn gives rise to a red (blue) shift of their respective resonances^18^. The propagation of the sound wave along the *x* axis of the PM leads to a time-dependent detuning of the two cavities


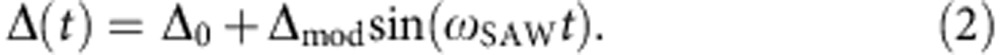


The amplitude of this modulation, 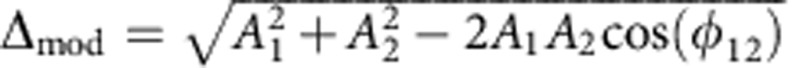, depends on the amplitudes of the modulations of the individual cavities, *A*_1_ and *A*_2_, and is maximum for *φ*_12_=180°. For Δ_mod_>Δ_0_, the detuning passes through zero, which results according to equation (1) in an avoided crossing of the normal modes. In Fig. 1d, the resulting temporal evolutions of the normalized mode frequencies (*ω-ω*_0_)/*J* obtained using equations (1) and (2) are evaluated over one acoustic cycle of *T*_SAW_ for a typical set of experimentally achievable parameters (*J*=1.2 Δ_0_, *A*_1_=1.5 Δ_0_, *A*_2_=1.9 Δ_0_ and *φ*_12_=162°). The dashed lines show the time evolution of the non-interacting (*J*=0) modes *M*_1_ (blue) and *M*_2_ (red). In strong contrast, the normal modes *M*_+_ and *M*_−_ start with initially *M*_1_-like (blue) and *M*_2_-like (red) single-cavity character, and develop to fully mixed symmetric (bonding) and antisymmetric (antibonding) character (green) at resonance. At this point, the coupling strength *J* of the PM can be deduced directly from the splitting of the avoided crossing. After traversing through the avoided crossing, the initial character of the modes is exchanged, with *M*_+_ and *M*_−_ possessing *M*_2_-like (blue) and *M*_1_-like character, respectively. Over the duration of one full acoustic cycle, the two modes are brought into resonance at two distinct times, giving rise to two avoided crossings, restoring the initial configuration. We performed full finite difference time-domain (FDTD) simulations to confirm and, in particular, quantitate the dynamic coupling. The evaluation for the used sample geometry is shown in Supplementary Fig. 2. We evaluate the calculated profiles of the electric field component in the plane of the PhC membrane and perpendicular to the SAW propagation (*E*_*y*_) in Fig. 1e. In the two left panels, the detuning is set to three times the coupling strength (Δ=3*J*), and thus the two modes remain well localized in one of the two cavities. For the resonance case, Δ=0, shown in the two right panels of Fig. 1e, the *E*_*y*_ exhibit the characteristic symmetric and antisymmetric superpositions for the *M*_−_ and *M*_+_ modes, respectively. For our sample geometry, the FDTD simulations predict *ℏJ*_sim_=0.71 meV. This value is set by the chosen separation, that is, barrier thickness, between the two cavities.

### Direct observation of SAW-controlled inter-cavity coupling

**Figure 2:**
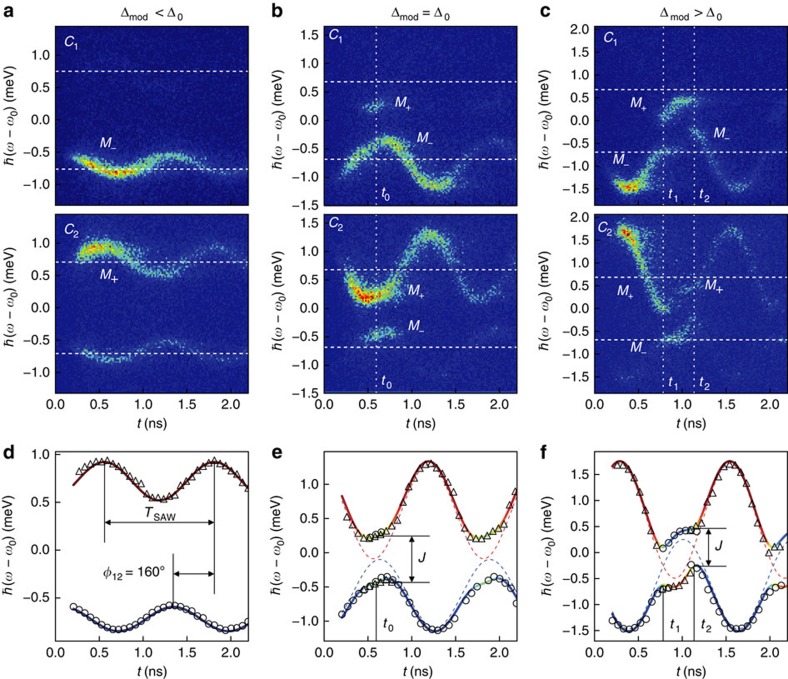
Experimental characterization for three different modulation amplitudes. (**a**–**c**) Time-resolved PL maps, measured on cavity *C*_1_ (upper panels) and *C*_2_ (lower panels). (**d**–**f**) Mode frequencies extracted from the measurements on *C*_1_ (circles) and *C*_2_ (triangles), fitted with the coupled mode model. For Δ_mod_<Δ_0_ (**a**,**d**), the modes are effectively decoupled. Their frequencies are modulated sinusoidally by the SAW with a phase difference of *φ*_12_=160°. For Δ_mod_=Δ_0_ (**b**,**e**), there is a time *t*_0_ at which the cavities are in resonance. At this point, the modes are split by the coupling strength *J* and they are delocalized over the PM, thus, both modes can be observed simultaneously on each cavity. When Δ_mod_>Δ_0_ (**c**,**f**), the modes exhibit an avoided crossing. This happens twice in each SAW cycle at times *t*_1_ and *t*_2_.

To experimentally verify the dynamic control of a PM by a *ω*_SAW_/2*π*=800 MHz SAW, we performed time-resolved spectroscopy with optical excitation at a well-defined time during the acoustic cycle. The emission of the PM was analysed in the time domain and as a function of relative emission frequency *ω*-*ω*_0_. We measured time-dependent emission spectra of the two cavities of PM1 for three characteristic modulation amplitudes. For a weak modulation, Δ_mod_<Δ_0_, shown in Fig. 2a, *C*_1_ and *C*_2_ show the expected phase-shifted sinusoidal spectral modulations centred (dashed horizontal lines) at their unperturbed resonances, which decay with a characteristic time constant of ∼1.3 ns. As the modulation is increased to Δ_mod_=Δ_0_ (Fig. 2b), the two single-cavity modes are brought into resonance at one distinct time *t*_0_=0.6 ns during the acoustic cycle. At this time, coherent coupling leads to the formation of bonding and antibonding normal modes. This directly manifests itself in the experimental data due to the emergence of new emission features stemming from the spatial delocalization of the bonding and antibonding modes. For the initially lower frequency cavity (*C*_1_), a new signal appears at the frequency of the normal mode *M*_+_, which was initially confined within the other cavity (*C*_2_). The initially higher-frequency cavity (*C*_2_) exhibits precisely the opposite behaviour, with the normal mode *M*_−_ appearing at time *t*_0_. The normal modes are split by the coupling strength *ℏJ*=(0.68±0.04) meV, very close to the value expected from our FDTD simulations. For further increased detuning Δ_mod_>Δ_0_, the two modes are brought into resonance at two distinct times, *t*_1_ and *t*_2_, during the acoustic cycle. After the first resonance at *t*_1_, coupling is suppressed, Δ>*J*, both modes are effectively decoupled and their single-cavity characters are exchanged compared with the initial configuration. The lower frequency mode *M*_−_, which is initially *C*_1_-like, is switched to *C*_2_-like character after *t*_1_, and vice versa. At the second resonance at *t*_2_, the system is reverted to its original configuration at the beginning of the acoustic cycle. This sequence of two time-offset coupling events gives rise to the experimentally observed anticorrelated time evolutions of the emission of *C*_1_ and *C*_2_. We extracted the time-dependent emission frequencies detected from the two cavities of PM1 and plotted them (*C*_1_ circles, *C*_2_ triangles) for the three modulation amplitudes in Fig. 2d–f.

From the set of measurements with varying tuning amplitude, we obtain mean values for the free parameters of the model, *ℏ*〈*J*〉_PM1_=(0.70±0.04) meV, 〈*A*_1_/*A*_2_〉_PM1_=0.75±0.05 and 〈*φ*_12_〉_PM1_=(155±10)°. Moreover, we note that the observed phase shift unambiguously confirms efficient coupling of the SAW into the sub-*λ*_SAW_ membrane. Its small deviation from the ideal value of 180° arises from the finite difference in the phase velocity of the acoustic wave within the membrane and the region of the transducer. The results from this model are plotted as lines and the character of the mode are colour coded. Clearly, our experimental data are in excellent agreement with the normal mode model detailed in the Methods section for all three modulation amplitudes. Both the time-dependent spectral modulation as well as the switching of the character of the modes are nicely reproduced.

### Statistical analysis and comparison with FDTD simulation

**Figure 3:**
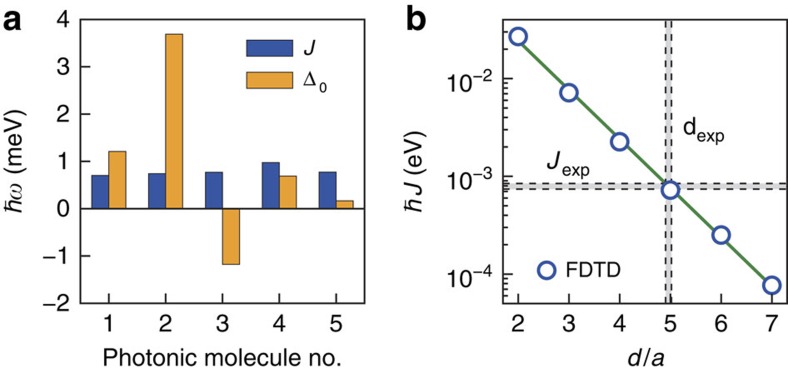
Statistical variation of PM properties and comparison with FDTD simulation. (**a**) Coupling strength *J* and static detuning Δ_0_ measured on five nominally identical PMs. (**b**) FDTD simulation of the coupling strength for different cavity separations *d*. The experimentally determined coupling strength is accurately reproduced by the simulation.

Such behaviour was experimentally confirmed for five different, nominally identical PMs for which we evaluated the mean of their respective key parameters. The experimentally observed static detuning Δ_0_ and coupling strength *J* are summarized in Fig. 3a. While the coupling strength, *ℏJ*=(0.8±0.1) meV, (blue) does not vary from PM to PM, the values of the static detuning, Δ_0_, shows a pronounced scatter ranging between −1.2 and +3.7 meV. These observations are in fact expected. *J* exponentially depends on the symmetric inter-cavity offset, *d*, and is thus robust and insensitive to small deviations from the ideal geometry due to fabrication imperfections. In strong contrast, the absolute resonance frequencies of the two cavities forming the PM are highly sensitive to these inherent and inevitable deviations from the nominal geometry. The resulting fluctuations of the cavity resonances reflect themselves in the observed variation of Δ_0_. Indeed, we observe pronounced static coupling (|*J*|≫|Δ_0_|) only for one single as-fabricated PM, labelled PM5. The corresponding experimental data are presented in Supplementary Note 1 and Supplementary Figs 3 and 4. The exponential dependence of *J* as a function of *d* is nicely confirmed by a best fit (line) to values calculated by FDTD (symbols) presented in Fig. 3b. The experimentally observed distribution of *J*_exp_ and the inter-cavity separation *d*_exp_ derived from *J*_exp_ and the FDTD simulation are indicated by the shaded horizontal and vertical bars. Clearly, the measured *J*_exp_ and its derived *d*_exp_ match perfectly the calculated value of *ℏJ*_sim_=0.71 meV and the nominally set *d*=5*a*. These narrow distributions centred around the calculated and nominal values are expected since *d* is large compared with typical imperfections in the nanofabrication.

## Discussion

In summary, we demonstrated dynamic optomechanical control of coherent interactions in a prototypical coupled nanophotonic system. When combined with optical nonlinearities, our tunable PM paves the way to dynamically controlled high-fidelity entanglement generation^38^ and distribution on a chip^39^,^40^. For larger switching rates, as required for Landau–Zener transition-based gates^9^, the underlying optomechanical coupling could be enhanced further by direct antiphased SAW excitation of localized vibronic modes of either nanocavity^35^. Since SAWs represent nanomechanical waves, Fourier synthesis allows to shape waveforms^34^. This paradigm in turn can be used to deliberately speed up or slow down switching as required for static or dynamic control schemes. Moreover, SAWs or SAW waveforms can be interfered generating one- or two-dimensional nanomechanical strain fields^41^. Thus, our approach could be directly scaled up to large arrays of coupled cavities^42^,^43^ or waveguides^44^. The coherent phononic nature of SAWs was verified by Metcalfe *et al*.^27^ who demonstrated resolved sidebands in the emission of a SAW-driven QD and performed optomechanical cooling and heating in this system. For the GaAs-based nanocavities, the cavity linewidth exceeds the sideband splitting set by *ω*_SAW_. Our approach can be readily transferred to silicon (Si)-based PhC membranes and optomechanical crystals using piezoelectric coupling layers^45^. On this platform, cavity photon loss rates of <1 GHz have been achieved^11^. Therefore, our approach is ideally suited to perform optomechanically driven all-photonic Landau–Zener-based entangling quantum gates with high fidelity, even for moderate SAW frequencies. The performance of this type of quantum gates can be drastically increased using higher SAW frequencies as demonstrated for instance by Tadesse and Li (ref. 25) and weaker cavity coupling rates. The exponential dependence of the coupling strength shown in Fig. 3b predicts a decrease from *J*∼170 to *J*<20 GHz when the offset is increased from 5*a* to 7*a*. This value of *J* is indeed compatible with state-of-the-art IDTs on Si (ref. 46). We note that our method is compatible with superconducting two-level systems and circuit electrodynamics. In particular, the geometry of transmon-type superconducting qubits matches that of IDTs. In a recent experiment by Gustafsson *et al*., these highly coherent two-level systems have been strongly coupled to single SAW quanta^47^. Finally we note that resonant frequencies of vibronic modes in optomechanical crystals^36^,^48^ match well with the frequency band covered by SAWs. The tight confinement of photonic and vibronic excitations in these structures gives rise to large optomechanical couplings^49^. In recent theoretical work, Ludwig and Marquart^50^ show a rich phase diagram for arrays of such optomechanical cavities, which delicately depends on the inter-cavity coupling. Thus, our method provides a tantalizing avenue to deliberately switch on and off the interaction or drive such systems all-mechanically.

## Methods

### Optical spectroscopy

For photoluminescence spectroscopy of the PM, the sample is cooled to *T*=5 K in a Helium-flow cryostat with custom-built-integrated RF connections. Off-resonant QDs are excited by an externally triggered diode laser emitting ∼90 ps pulses at a wavelength of 850 nm, which is focused to a 1.5-μm spot by a NIR × 50 microscope objective. The train of laser pulses is actively locked to the RF signal exciting the SAW to ensure optical excitation at an arbitrary but well-defined time during the acoustic cycle.^51^ The emission from the sample is dispersed by a 0.75-m imaging grating monochromator. A fast (<50 ps rise time) Si single-photon avalanche detector is used for time-resolved single-channel detection of the spectral modulation of the optical modes^52^. Details of the phase-locked excitation scheme and additional data on single-cavity measurements are discussed in Supplementary Note 2 and Supplementary Fig. 5.

### Sample structure

We start by fabricating the PhC membranes from a semiconductor heterostructure grown by molecular beam epitaxy (MBE). This heterostructure consists of a 170-nm GaAs layer with self-assembled InGaAs QDs at its centre, on top of a 725-nm-thick Al_0.8_Ga_0.2_As sacrificial layer. The PM structure is defined by electron beam lithography and transferred into the heterostructure by inductively coupled plasma reactive ion etching (ICP-RIE) etching. In a wet chemical etching step using hydrofluoric acid, we removed the sacrificial layer to release a fully suspended membrane. The PMs are deliberately designed to be off-resonant with the QD emission to achieve a sufficiently long decay time of the cavity emission. The cavity resonance is detuned by several meV from the QD emission band. Thus, no Purcell-enhanced spontaneous emission with fast radiative rates occurs^53^. In turn, the cavity mode emission arises from phonon- or Coulomb-assisted feeding mechanisms on a ∼1-ns timescale^54^,^55^,^56^. These long timescales set the experimental time window over which the cavity mode can be detected using time-resolved photoluminescence spectroscopy. Additional data of time-resolved experiments of the cavity emission are shown in Supplementary Fig. 5d.

IDTs were defined using electron beam lithography and metallized with 5 nm Ti and 50 nm Al in a lift-off process. The finger period is 3.83 μm, resulting in a resonance frequency of 800 MHz at the measurement temperature of 5 K. The IDTs consist of 80 pairs of 185-μm-long fingers and are located at a distance of 800 μm from the array of PhCs. The PhCs are arranged in a staggered pattern, that is, offset in the direction perpendicular to the SAW propagation. This arrangement ensures comparable SAW coupling to each PhC device. A schematic of this layout is included as Supplementary Fig. 6.

### Coupled mode model

We treat the PM as a model system of two coupled cavities. The single-cavity modes *M*_1,2_ have the complex amplitudes *a*_1,2_ and the frequencies *ω*_1,2_. In the presence of finite coupling and the absence of dissipation, the time evolution is given by


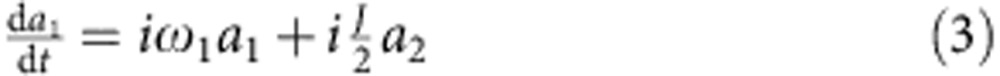


and


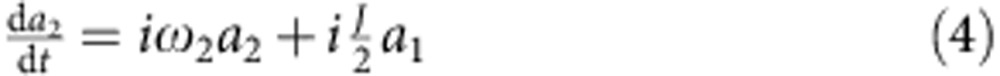


with a real coupling constant *J*.

The resulting normal modes have the frequencies given by equation (1) 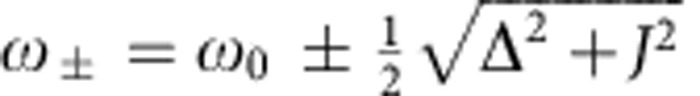 with centre frequency 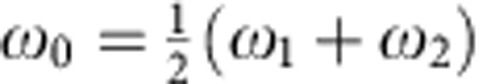 and detuning Δ=*ω*_2_-*ω*_1_. We note that since *J*>0, the low-frequency mode, *ω*_−_, and the high-frequency mode, *ω*_+_, correspond to symmetric and antisymmetric superpositions of the uncoupled, single-cavity modes, respectively.

## Additional information

**How to cite this article:** Kapfinger, S. *et al*. Dynamic acousto-optic control of a strongly coupled photonic molecule. *Nat. Commun.* 6:8540 doi: 10.1038/ncomms9540 (2015).

## Supplementary Material

Supplementary Information Supplementary Figures 1-6, Supplementary Notes 1-2 and Supplementary References
