# Supplementary material for: Dynamic acousto-optic control of a strongly coupled photonic molecule
Source: Nat Commun. 2015 Oct 5;6:8540. doi: 10.1038/ncomms9540 (PMC4600751; doi:10.1038/ncomms9540)
Supplement: Supplementary Information — Supplementary Figures 1-6, Supplementary Notes 1-2 and Supplementary References [file ncomms9540-s1.pdf]

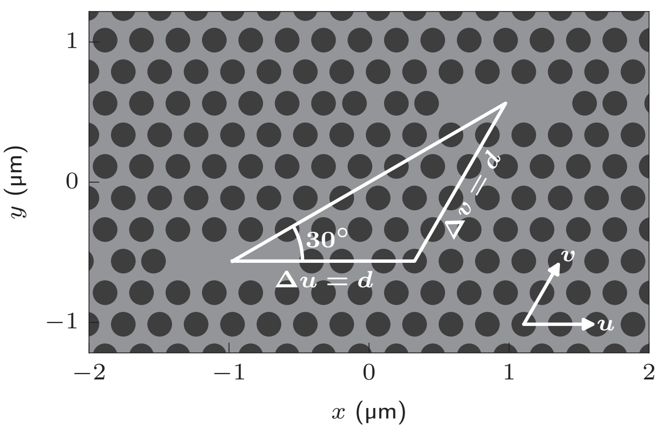

Supplementary Figure 1. *Photonic molecule geometry* – The two cavities are separated by distances $\Delta u = \Delta v =: d$ along the principal axes of the photonic crystal lattice. In this configuration the axis connecting the cavities forms an angle of 30° with the u-axis. For this arrangement, the intercavity coupling is expected to be maximum [1]. For the investigated PMs we used a separation of $d = 5a$ ($a = 260\,\text{nm}$), for which FDTD simulations predict a coupling strength of $J_{\text{sim}}/2\pi = 170\,\text{GHz}$.

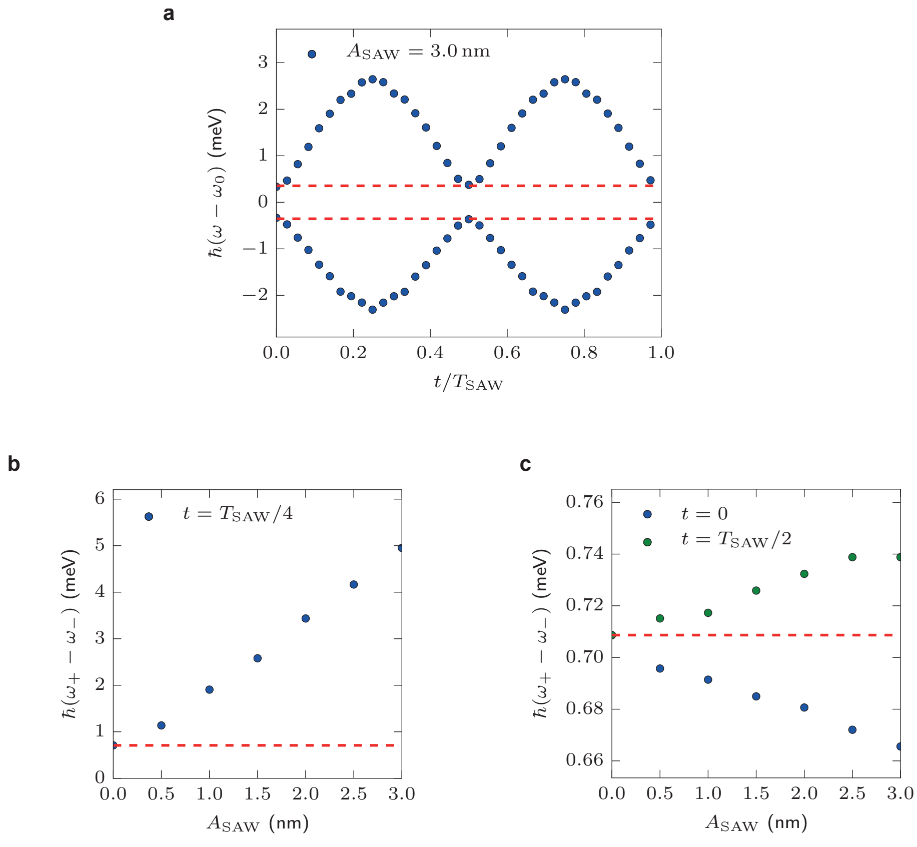

Supplementary Figure 2. *FDTD Simulations of SAW tuning* – The simulations were performed with $\Delta_0 = 0$ and $\phi_{12} = 180°$. (a) Evolution of the normal modes over one acoustic cycle. The modes show avoided crossings at $t = 0$ and $t = T_{\text{SAW}}/2$ with a normal mode splitting (indicated by the red dashed lines) of $J = 170\,\text{GHz}$. (b) Splitting of the detuned modes at the point of maximum detuning ($T_{\text{SAW}}/4$) as a function of the SAW amplitude A_{SAW}. The splitting approaches J for $A_{\text{SAW}} = 0$ and becomes a linear function of A_{SAW} at high detunings. This is in agreement with Eq. 1, as the detuning Δ depends linearly on A_{SAW} [2]. (c) At finite SAW amplitudes, the simulations predict a deviation of J from the static value. An increase (decrease) is expected for $t = 0$ ($t = T_{\text{SAW}}/2$), which corresponds to a reduced (increased) inter-cavity separation for the minimum (maximum) of the SAW located in between the two cavities. As the resulting deviation is below our experimentally achievable resolution, the treatment of J as constant is fully justified. However in systems with improved quality factors, temporal and spectral resolution, and signal levels this effect could become resolvable.

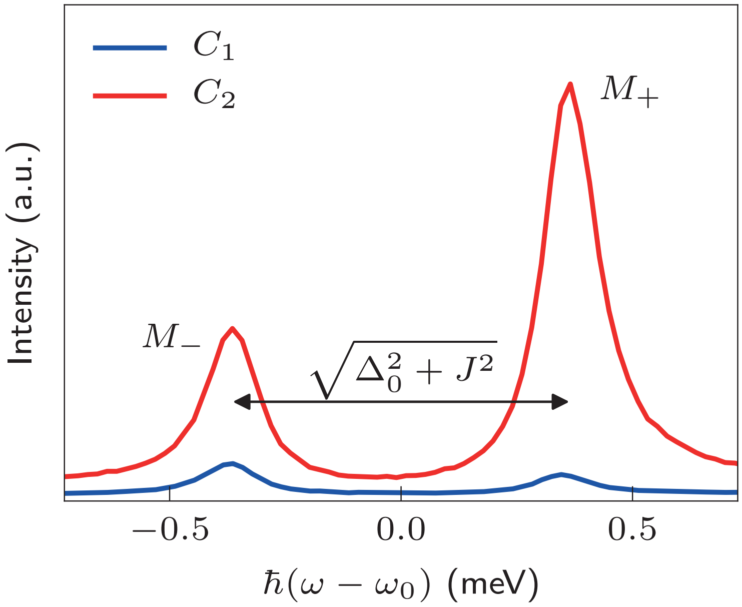

Supplementary Figure 3. *Unperturbed emission spectra of PM5* – Emission spectra of a PM (labeled PM5 in the main paper) which has vanishing static detuning $\Delta_0 \approx 0 \ll J$. Since this PM exhibits coupling in the static state, both normal modes can be observed on each cavity. The difference in intensity between cavity C_1 and C_2 can be attributed to different excitation or extraction efficiencies.

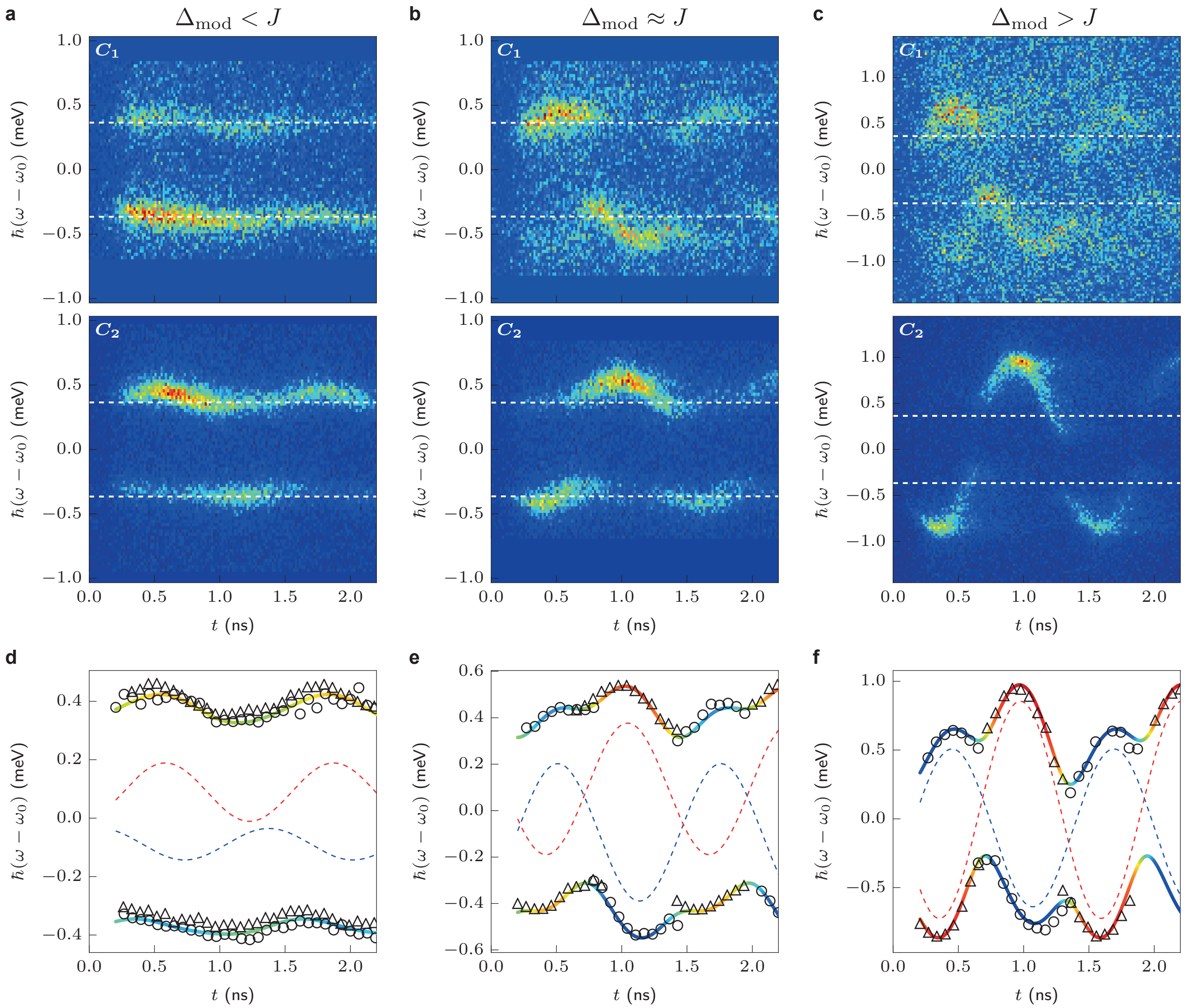

Supplementary Figure 4. *SAW-tuning of PM5* – Time resolved characterization of PM5 for different modulation amplitudes. (a–c) Time resolved PL maps, measured on cavity C_1 (upper panels) and C_2 (lower panels). (d–f) Mode frequencies extracted from the measurements on C_1 (circles) and C_2 (triangles), fitted with the coupled mode model described in the main paper. Increasing Δ_{mod} leads to a more pronounced single cavity character between the avoided crossings. For the parameters of this PM we obtain the mean values $\langle J \rangle_{\text{PM5}} = 190\,\text{GHz}$ and $\langle \Delta_0 \rangle_{\text{PM5}} = 40\,\text{GHz}$.

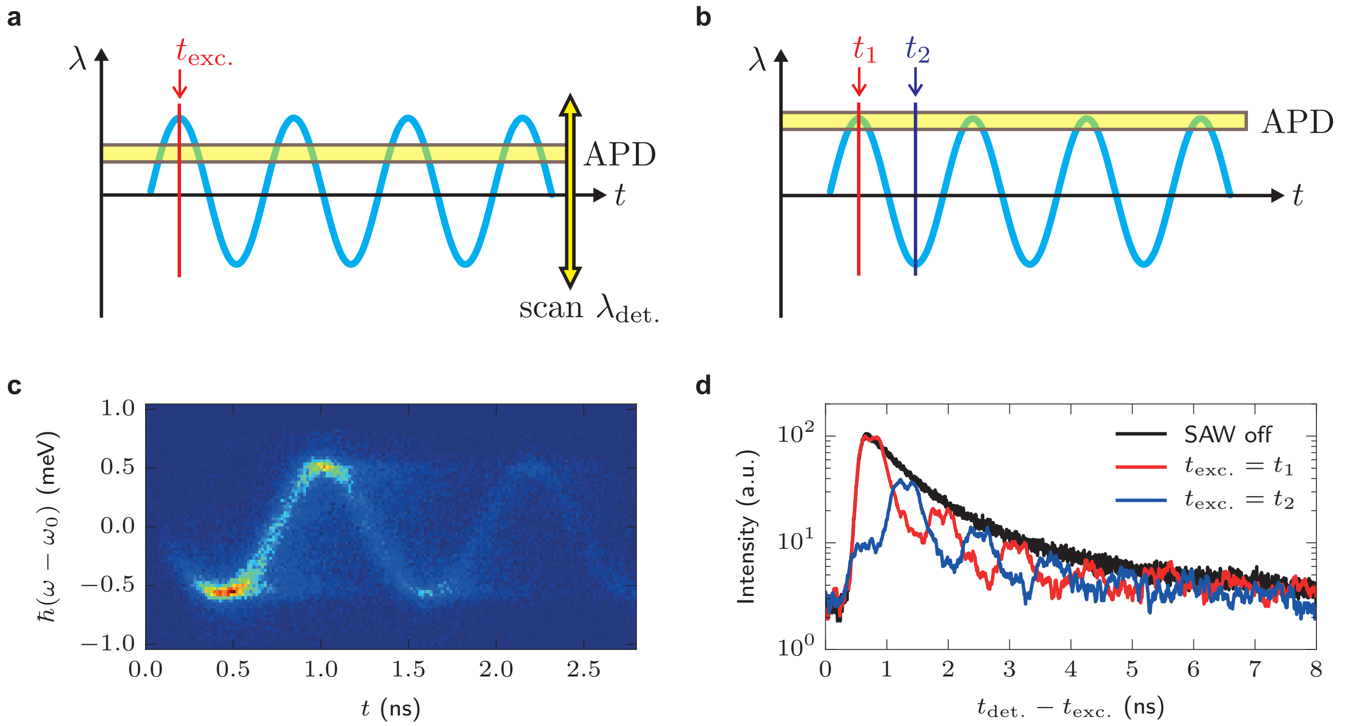

Supplementary Figure 5. *Single cavity tuning experiments using two different excitation and detection schemes* – (a) Experimental scheme as applied throughout the paper with the excitaiton time t_{exc} being fixed and the detection wavelength λ_{det} being scanned. (b) Complementary experimental scheme with λ_{det} being fixed and t_{exc} being varied. (c) Single cavity tuning by a SAW monitored using the scheme shown in (a). (d) Time transients recorded at two distinct excitation times as shown in (b). The black transient is the unperturbed cavity decay which envelops the SAW modulated transients.

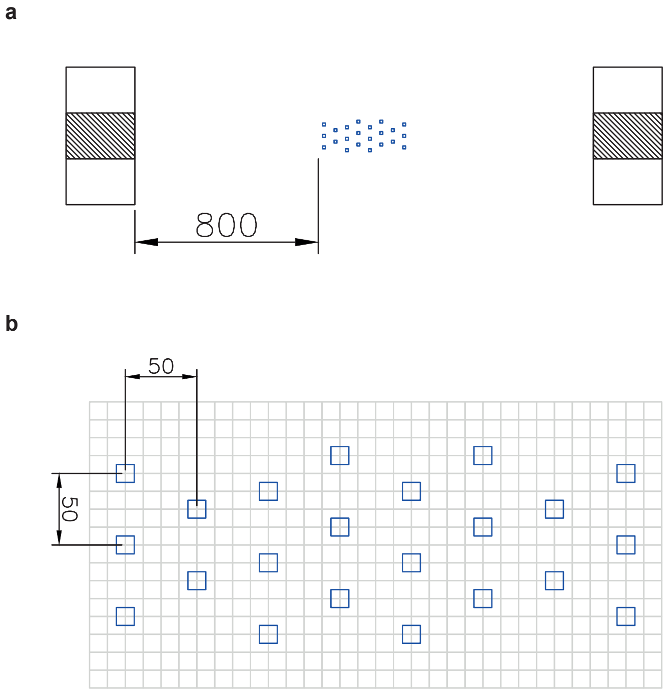

Supplementary Figure 6. *Sample layout* – (a) The array of PhC devices is centered between to IDTs at the left and the right. (b) Zoom to PhC device array. All distances are in microns.

SUPPLEMENTARY NOTE 1: PHOTONIC MOLECULE WITH $\Delta_0 \approx 0$

For PM5 signal from both supermodes are detected from each cavity as can be seen from the unperturbed emission spectra in Supplementary Figure 3. Since $J \gg \Delta_0$ the modulation amplitude Δ_{mod} has to be compared to J for PM5. This is in contrast to PM1 (presented in the main paper) and PM2-PM4, for which $J < \Delta_0$ and Δ_{mod} has to be compared to Δ_0. In addition, the time interval between the two avoided crossings is increased. In Supplementary Figure 4 we present a full set of experimental data of PM1. These expeiments are performed analogously to that on PM1 shown in Fig. 2 of the main paper. From the data in Supplementary Figure 4 (c) and (f) we determine temporal offsets of 660 ps and 580 ps between the first and second, and the second and third avoided crossing, respectively.

SUPPLEMENTARY NOTE 2: SINGLE CAVITY TUNING EXPERIMENTS USING TWO DIFFERENT EXCITATION AND DETECTION SCHEMES

In Supplementary Figure 5 we present details on the phase-locked and time-resolved photoluminescence spectroscopy employed in our experiments. All data were obtained from a *single* nanocavity modulated at $\omega_{\mathrm{SAW}}/2\pi = 850\,\mathrm{MHz}$. In panels (a) and (c) we present a schematic of the experimental scheme and data, respectively. This scheme was also employed to obtain the data shown throughout the paper and in Supplementary Figure 4. As shown in Supplementary Figure 5 (a), the laser excites the system at an arbitrary, but well-defined time during the acoustic cycle, $t_{exc.}$. The emission of the nanocavity is detected in the time domain as a function of the detection wavelength λ_{det}. In our experiments, this was achieved by scanning the monochromator and recording for each wavelength a time transient with an avalanche photodiode (APD). The such obtained time transients are plotted color coded as a function of time delay after the laser excitation. The result obtained from a single cavity is shown in Supplementary Figure 5 (c). In Supplementary Figure 5 (b) and (d) complementary scheme is employed. As shown schematically in Supplementary Figure 5 (b), the detection wavelength is kept fixed, but the time of photoexcitation was set to two different values. At t_1, the cavity mode is tuned to λ_{det}, while for $t_2 = t_1 + \pi/\omega_{\mathrm{SAW}}$. Thus, for excitation time t_2, the cavity mode is initially detuned from λ_{det} and becomes resonant again at half a SAW period later. The two time transients recorded for photoexcitation at t_1 and t_2 are plotted in Supplementary Figure 5 (d) as red and blue lines, respectively. Both transients show a clear modulation with the SAW's period, T_{SAW}. The two modulations are precisely offset by $|t_2 - t_1| = \pi/\omega_{\mathrm{SAW}} = T_{\mathrm{SAW}}/2$. Moreover, they are enveloped by the unperturbed cavity decay (recorded at the unperturbed resonance wavelength) plotted in black.

SUPPLEMENTARY REFERENCES

[1] Chalcraft, A. R. A., Lam, S., Jones, B. D., Szymanski, D., Oulton, R., Thijssen, A. C. T., Skolnick, M. S., Whittaker, D. M., Krauss, T. F. & Fox, A. M. Mode structure of coupled L3 photonic crystal cavities. *Opt. Express* **19** 5670-5675 (2011).

[2] Fuhrmann, D. A., Thon, S. M., Kim, H., Bouwmeester, D., Petroff, P. M., Wixforth, A. & Krenner, H. J. Dynamic modulation of photonic crystal nanocavities using gigahertz acoustic phonons. *Nature Photon.* **5**, 605-609 (2011).
